# Mr. Kirby on the Power, Wisdom, and Goodness of God, in the Creation of Animals

**Published:** 1836-01-01

**Authors:** 


### On the Power, Wisdom, and Goodness of God in the Creation of Animals, and in their History, Habits, and Instincts. By the Rev. *W. Kirby*, M.A. F.R.S. &c.

In our last number, we endeavoured to give a concentrated *coup d'œil* of Mr. Kirby's first volume, closing our sketch with the subject of vermes. The second volume, or thirteenth chapter, opens with *cirripedes* and *crinoïdeans.* The *first* of these, denominated *lepas* by Linné, and known in this country by the general name of *barnacles,* have a soft body, protected by a multivalve shell—are without eyes, or any distinct head—have no locomotion, but can fix themselves to various substances. Their body, which has no articulations, is enveloped in a kind of mantle, and has numerous tentacular arms, fringed on each side, and issuing by pairs from jointed pedicles. The mouth is armed with transverse toothed jaws, and furnished with feelers. They have a spinal chord, gills for respiration, and a heart and vascular system.

"These animals roll up and unroll their arms with great velocity, thus creating a little whirlpool, that brings to their mouth an abundant supply of animalcules, an action which Poli compares to fishermen casting a net." 3.

The functions and instincts of the cirripedes are little known—" of this we are sure—that they work HIS work who gave them being, and assigned them their station in the world of waters."

### CRINOÏDEANS.

In the deepest abysses of the ocean, there probably lurks a tribe of plant-like animals, abounding in genera and species (judging from their fossil remains) which are rarely seen in a recent state. Only three or four specimens of these animals have been taken—and one of these is in the Hunterian Museum in London. They differ from the cirripedes in having no jaws, and, therefore, their food, whatever it is, must be animalcular or liquid.

The 14th chapter brings us to a "great branch of the animal kingdom, which, in its higher tribes, exhibits Divine Wisdom, acting in and by the instincts of creatures, small, indeed, in bulk, but mighty in operation, in a way truly admirable." This group is the—

### CONDYLOPES.

The distinctive characters of this class or group are easily given. The animal is locomotive—body consists of two or more segments—legs jointed. This section of the animal kingdom has been subdivided by Cuvier and Latreille into three classes—crustaceans, arachnidans, and insects. Dr. Leach, taking the respiratory organs for his guide, begins with three primary sections—those that have *gills*—those that have *sacs*, and those that have *tracheæ*. Out of these he forms five classes—crustaceans, arachnoidans, acarines, myriapods, and insects. Our author finds it exceedingly difficult to determine the class which ought to be regarded as forming the first step in an ascending series. He begins, however, with the *crustaceans*. Like the infusory animalcules, these form a kind of centre, sending forth rays to different parts, some inclosed in a bivalve shell, others assuming more of the crustacean form, &c. There is no one character common to the whole of this remarkable class. Generally, however, they are covered, not by a calcareous and solid, but by a horny and thin integument. One group of them lives by suction, and is parasitic upon other aquatic animals—most of them, however, masticate their food, but without the aid of maxillary legs. This class our author divides into two orders, according as they inhabit fresh and salt water—the *Branchiopods* and *Pœcilopods.* The first order mostly frequent stagnant pools, where they move about with great rapidity. They are regarded as predatory, and to make the infusory animalcules their prey. Some of them are supposed to be herbivorous. Like the infusories, they have the power of revivescence, after death by the drying up of the waters in Summer. Latreille thinks that some of them have the power, after drawing in all their organs, of hermetically sealing their shells till the return of moisture! It is in this class of animated beings that we first discover the remarkable phenomenon of metamorphosis or moulting. These creatures fix themselves to some substance at hand, and then move about their limbs and the valves of their old shells till they loosen and cast off their exuviæ. These moults follow each other at an interval of five or six days, and it is not till after the third moulting that the animal acquires the reproductive faculty. Hitherto form after form has appeared on the stage of animal existence, each distinguished by characters indicating an elevation in rank and station; but *here* we are got among animals which, at different periods of their existence, assume a higher tone of character, and become endued with organs calculated for a more extended range. Sometimes from being purely aquatic, the animal (cyclops for example) becomes a denizen of the earth and air—or of air, earth, and water at the same time! It is doubtful whether the higher orders of the crustaceans undergo a real metamorphosis, or only change their shells annually. Insects, we know, do not increase in size after their last change.

"Do not these successive changes in the outward form, functions, and locomotions of so many animals, preach a doctrine to the attentive and duly impressed student of animal forms, and their history—do they not symbolically declare to him, that the same individual may be clothed with different forms, in different states of existence, that he may be advanced, after certain preparatory changes, and an intermediate interval of rest and repose, to a much more exalted rank; with organs, whether sensiferous or locomotive, of a much wider range; with tastes more refined; with an intellect more developed, and employed upon higher objects; with affections more spiritualized, and further removed from gross matter?" 27.

The power of multiplying in these animals is almost incredible. It is calculated that one female cyclops, after one fecundation, (which serves for several successive generations) will, in six weeks, prove to be the progenitrix of *four thousand five hundred millions* of other cyclops!! An animal of the present order was observed by Captain Koetzebue in such myriads, that the sea exhibited a red stripe, a mile long, and a fathom broad, produced by a species which individually was scarcely visible! These powers of multiplication, however astonishing to us, are, no doubt, given to these creatures for wise purposes. They supply food, in abundance, to a variety of other creatures, both above and below themselves on the scale of existence. In turn, they prey upon other and much more powerful animals. The gigantic whale, the sagacious dolphin, the terrific shark cannot defend themselves against these minute parasites.

The next order includes all the *marine entomostracans,* and is quickly dismissed by our author. The first section consists of a single genus, typified by the Monoculus Polyphemus of Linné. In the West Indies it is well known as the King-crab. Like the cirripides, they have no distinct head; but they have a long angular tail, simple and compound eyes, &c. Some of them, in the eastern seas, grow to two feet in length. Its most remarkable part is the tail, shaped like a stiletto, and so sharp at the extremity, that it will easily pierce the body of almost any animal with which it comes in contact. Its legs are armed with pincers, and it is evidently predaceous upon small animals. The second section differ from the others by the manner of taking their food. They are parasitic upon the cetaceans, fishes, some reptiles, and crustaceans, whose juices they imbibe by *suction.* They often attach themselves to the gills of these animals.

### Crustacean Condylopes.

We are now come to a class where the organs of locomotion assume a more perfect construction, in some measure resembling those of the vertebrated animals. The development of the locomotive organs, from the lowest to the highest order of animals, will form the subject of a distinct chapter in a future part of the work, and therefore we shall very briefly touch upon it here.

The anterior legs are strictly *arms,* terminating in a dedactyle hand, furnished with a kind of finger and thumb, by which it seizes its prey. This is the *chela* or claw of the lobster and crab. This structure is particularly fitted to their wants and situations. A hand like ours would be an incumbrance rather than an useful instrument of prehension, where great strength is concentrated into two fingers instead of being distributed over five. In some of the crustaceans, however, the claws are very small; but this is made up by number, and though they cannot lay hold of large animals, they can seize, at the same moment, several small ones. The shrimp, prawn, pandle, &c. afford examples.

Aristotle, long ago, noticed a crab found in Phœnicia, under the name of the horseman (Hippous) which he said ran so fast that it was not easy to catch him. Ollivier found that this account was true—at least of those he saw on the coast of Syria—and Bosc found a species in Carolina which he could scarcely overtake on horseback, and shoot with a pistol. Another kind of land-crab has a most extravagantly large claw on one side, and a very diminutive one on the other. When he retires into his hole, he leaves the large claw in the passage, to prevent the entrance of intrusive visitors. They will dispute a carrion with vultures. They only enter the water to lay and hatch their eggs. There is another kind that remain in burrows for months together, and are exceedingly prolific, though preyed upon by numerous animals, as otters, bears, birds, tortoises, &c. The Hermit-crabs have naked abdomens, unprotected by any hard crust; but God has given them a compensating instinct. They search about till they find a univalve shell fitted to their size, which they make their habitation and carry about with them. They thus, as it were, clothe themselves with the cast-off garments of other animals. This is the first instance we find in the creation of physical hypocrisy—of *assuming* the aspect of a different animal—perhaps one that is known to be inoffensive, in order that the assumer may deceive the unwary, and entrap his victim! There is a genus of crabs or rather lobsters which quit the sea at night, and ascend the cocoa-nut and other palm trees, to seize the fruit. This species is gigantic. Among the larger species is a ferocious animal, the *thorny lobster* of the London market, which is sometimes nearly three feet in length. It is also called the *cray-fish.* The common lobster, so well known to both the ancients and the moderns, we may pass over. All crustaceans cast their crust annually—which seems a most wonderful operation, considering the density of the incrustation covering not only the head, trunk, and abdomen, but even the limbs! These animals live sometimes twenty years or more, and keep increasing in size during the greater part of their lives. The mode of casting their crusts was unknown till discovered by the French naturalist—Reaumur. It is very curious. We shall extract the description of it.

"He observed that when one of these was about to cast its crust, it rubbed its feet one against the other, and gave itself violent contortions. After these preparatory movements, it swelled out its body more than usual, and the first segment of its abdomen appeared more than commonly distant from the thorax. The membrane that united them now burst, and its new body appeared. After resting for some time, it recommenced agitating its legs and other parts, swelling to the utmost the parts covered by the thorax, which was thus elevated and separated from the base of the legs; the membrane which united it to the underside of the body burst asunder, and it only remained attached towards the mouth. In a few minutes, from this time, the animal was entirely stripped except the legs. First the margin of the thorax was seen to separate from the first pair of legs; at that instant, drawing back its head, after reiterated efforts, it disengaged its eyes from their cases, and all the other organs of the anterior part of the head; it next uncased one of its fore legs, or all or part of the legs of one side, which operation is so difficult that young ones sometimes die under it. When the legs are disengaged, the animal casts off its thorax, extends its tail briskly, and pushes off its covering and that of its parts. After this last action, which requires the utmost exertion of its remaining vigour, it sinks into a state of great weakness. Its limbs are so soft that they bend like a piece of wet paper; but if the back is felt, its flesh appears unexpectedly firm, a circumstance arising, perhaps, from the convulsive state of the muscles. When the thorax is once disengaged, and the animal has begun to extricate its legs, nothing can stop its progress. Reaumur often took them out of the water with the intention of preserving them half uncased, but they finished, in spite of him, their moult in his hands. Upon examining the exuviæ of these animals, we find no external part wanting; every hair is a case which covers another hair. The lower articulations of the legs are divided longitudinally at a suture which separates during the operation, but which is not visible in the living animal." 54.

The great crustacean host, of which we probably do not know half the species, is a most valuable gift to mankind, as well as to the various inhabitants of the waters—varying, as they do in size, from the great thorny lobster down to the minute tribes of entomostracans! Their power of multiplication defies diminution of their number by any ratio of consumption.

We must pass almost entirely over the XVIth Chapter on Mynapod Condylopes, though it contains many curious observations on the locomotive powers of this class of creatures, which we recommend to the reader. We shall merely allude to the *suckers*, which bear the same relation to the cephalopods that the hand or foot does to the higher animals. When the animal means to use this curious apparatus it applies its sucker flat to whatever body it wishes to adhere to. It then contracts its sphincter which produces a cavity or vacuum in the centre. By this contrivance it attaches itself to the foreign body with a force proportioned to the diameter of the sucker, and to the weight of the column of water or air of which it is the base. This force being multiplied by the number of suckers, it is easier to tear the legs off the animal's body, than separate them from the surface to which they are attached.

It is not a little curious that some animals, which have no *organs* whatever for locomotion, have yet the *power* of transporting themselves to the most distant regions of the unfathomable ocean! The barnacle, for example, which is unprovided with any apparatus for motion, and, therefore, would appear to be destined to remain, during its life, in the spot where it was first created, can, by means of its sucker, take its passage on the back or belly of the whale, dolphin, shark, or other rover of the deep, and traverse thousands of miles without expence or trouble. As it does not draw nutriment from the animal which it rides, it quits its grasp when hungry, and floats about or sinks to the bottom in search of food, till inclined to make another excursion to some distant region of the deep!

There are but few of the vertebrated animals that are provided with suckers. The genus GEEKO, however, is one of them. Their suckers consist of transverse laminæ, occupying the terminal part of the toes. By means of these, they can walk against gravity, and thus pursue insects up perpendicular, or along prone surfaces. By these, they can mount the polished chunam walls of houses in India, or climb the trees of the West India Isles!

The author has made many ingenious remarks on the wings of insects, and some fishes (as the flying fish), and their comparison with the wings of birds. But we must pass over these, and numerous other subjects of great interest, in order to come to the prominent feature of the work, viz.

### INSTINCT.

What is INSTINCT? It is more easily understood than defined. It is not easy to dissipate all the darkness that envelops the *secondary* or intermediate *cause* of instinct. Could even the bee and the ant tell us what it is that goads them to their several labours, and instructs them how to perform them, we would still have little reason to cry EUREKA! One thing is certain, that this mysterious impulse diminishes, as reason or intellect becomes developed in man and the higher animals, while, as we descend in the scale, we come to tribes that exhibit, in the most miraculous manner, the workings of a divine power, performing operations that the intellect and skill of MAN would in vain attempt to rival or imitate!

The ancients had no, or very obscure notions of instinct. Aristotle himself resolves motion into intellect and appetite. He speaks of the hive-bee, but says not a syllable as to whether he attributes their wonderful proceedings to any faculty distinct from intellect. In one place he makes use of the following remarkable expression. "Some of the animals that have no blood, have a more *intelligent soul* than some of those that have blood, as the bee and the ant genus."* From this, indeed, it is clear, that the stagyrite drew no distinction between intellect and instinct—or rather that the

* De Part. Animal. L. ii. cap. 4.

latter was the perfection of the former. We quite agree with our author, that instinct appears to be the direct gift of the Creator, and serves as a command, from which the animal cannot swerve. Intellect is also the gift of the Almighty; but man can use it or abuse it as he likes. We would illustrate our ideas of intellect and instinct, by a comparison of the cerebro-spinal with the ganglionic nerves and functions. The former are under the command of the will—at least the motions to which they give origin—the latter, or ganglionic nerves, perform their functions without any will or command—without even the knowledge of the individual. The limits, however, between intellect and instinct are not so easily fixed, some animals, as the dog, the elephant, and the monkey exhibiting no trifling degree of reason, superadded to instinct. In one respect, instinct is superior to reason —namely, because it never errs, whereas the boasted intellect of man is constantly deviating from the rule of right. Mr. Kirby, after some prefatory observations respecting the *interference* of the Deity, in changing or suspending his own laws (observations which are far from increasing our respect for his own judgment), proceeds to an explanation of "the proximate cause of instinct." He says that this must be either metaphysical or physical—or a compound of both characters.

1. "If *metaphysical,* it must either be the *immediate* action of the Deity, or the action of some *intermediate* intelligence employed by him, or the *intellect* of the animal exhibiting it.

2. If *physical,* it must be the action or stimulus of some physical power or agent employed by the Deity, and under his guidance, so as to work His will upon the organization of the animal, which must be so constructed as to respond to that action in a certain way; or by the exhibition of certain phenomena peculiar to the individual genus or species.

3. If *compound* or *mixed,* it will be subject occasionally to variations from the general law, when the intelligent agent sees fit." 230.

We hope our readers are more fortunate than ourselves, in finding any thing in the foregoing definition—beyond that which is said to be the legitimate object of language—the concealment of our thoughts! The first hypothesis was supported by Addison, and means, if it have any meaning, that "God is the soul of brutes." This idea of an anima mundi, however, is considered by our author as unworthy of the Creator—and, moreover, that it is erroneous, because INSTINCT does sometimes err, which would not be the case were God the soul.

"For, if God were their *immediate* instructor, would it be possible for the flesh-fly, as I have seen that she does, to mistake the blossom of the carrion-plant* for a piece of flesh, and lay her eggs in it; or for a hen to sit upon a piece of chalk, as they are stated to do,† instead of an egg? Still all instincts are from God, He decreed them, and organized animals to act according to that decree, and employed means to impel them to do so." 231.

We think there is a metaphysical subtlety in the foregoing passage which is far from convincing. If instincts be from God, and God has decreed them, their occasional liability to err—or rather to be deceived, is as derogatory to the Deity as if he himself was the direct agent. Besides, we do

* Stapelia hirsuta.

† Spectator, ii. n. 120.

not think that the instinct of the hen is in error, because a piece of chalk so resembles an egg as to deceive her. If she went and sat upon a piece of hot iron, we would say decidedly that her INSTINCT was as much at fault as REASON is, when man rushes upon his sword. When the birds pecked at grapes, chiselled out of the marble by the ancient sculptor, it was never said that their *instinct* was in fault, but only their *perception* !

It appears that Mr. French is the author of the other hypothesis—the *intermediate* agency of the Deity as the cause of instinct—in other words, that angels and demons are employed in urging animals (man we should suppose included) to good and bad actions. We shall not waste any time upon this brilliant hypothesis, and, to do justice to Mr. Kirby, he discountenances this doctrine, though he deems it worth his while to combat it.

He next considers "whether instinct be the result of the intellectual powers of the animal"—and concludes in the negative, because, in fact, the more intellect, the less instinct do we find throughout the great chain of animated beings. The following passage is sufficient answer to the mistakes of the hen and the flesh-fly.

"But when we reflect further, that even in cases where the instincts are most complex and wonderful, the animal practises them *infallibly*, without guide or direction, and is as expert at them when it first emerges into life, as when it has been long engaged in the practice of them ; it follows that it must be instructed in them from the first moment of its existence in the state in which it exercises them, by an infallible teacher. The bee, the moment it emerges from the pupa, begins to collect honey and pollen, and to perform all the other manipulations that belong to her instincts." 237.

In the higher animals, it is true, the case is different. They emerge into life in a state of helplessness that requires, as in man, a temporary sojourn under the protection of the parent. These have more of intellect and less of instinct than the bee—but the *plus or minus* does not constitute an essential difference in the kind of instinct. This *storge* stimulates the parent to take care of the young, till the young is able to provide for itself, when the link of connexion between parent and progeny is broken for ever.

"This is, therefore, chiefly instinctive; but in the most intellectual of all animals, where instinctive love ceases, rational love begins ; and care and anxiety for the welfare of our offspring, and affectionate regard for their persons, continues after they cease to have any need of our help and attention." 238.

We wish we could flatter ourselves, that here we have a well-defined line of demarcation between man and the inferior animals. But whoever contemplates the difference of attachment between the mother and child, when the latter is young, and when both are old, must acknowledge that the bond of union is strong as animal instinct in the one case, and all but null in the other!

Our author candidly acknowledges, that "animals of the higher grades, by means of their organs of sensation, acquire ideas upon which they in some sort *reason*, by comparing one with another. Thus they get experience, and, as they grow older, literally grow wiser." This is, we fear, as much as we can say for the great mass of mankind ! It is more, indeed, than we can say for multitudes of our fellow-creatures ! If it be admitted, it deprives us of almost every distinction between man and animals, except in the *plus or minus* of reason—and REVELATION.

The following passage we cannot comprehend.

"With regard to truly *instinctive* actions, they invariably follow the *development of the organization;* are neither the result of instruction, nor of observation and experience, but the action of some *external agency* upon the organization, which is fitted by the Omniscient Creator to respond to its action." 238.

Now, if instinct invariably follows the *development* of organization, the legitimate inference is, that this development is the cause of the instinct. What is instinct, but the aggregate functions of the animal. Digestion, the function of the stomach, is instinctive. The Creator endowed the structure of every animal, and every part of an animal, with its peculiar function or instinct, and we cannot see any necessity for further interference of the Divine Architect, after the endowment was once given. We do not think that instinct, then, is "an *external* agency *upon* the organization," but that it is, as we have said, the function or endowment of the organization, coeval with the structure, and willed by the omnipotent Architect.

We do not find any thing very definite or intelligible in the conjecture of our author, at page 242, where he imagines that "the great physical powers of the Heavens," those vicegerents of God, "may be the *inter-agents* by which the Deity acts upon animal organizations and structures, to produce all their varied instincts." This is surely explaining the "ignotum per ignotius." The following is very harmless and very amusing—but no more.

"An eminent French zoologist* has illustrated the change of instincts, resulting from the modification of the nervous system, which takes place in a butterfly, in the transit to its perfect or imago state from the caterpillar, by a novel and striking simile. He compares the animal to a portable or hand organ, in which, on a cylinder that can be made to revolve, several tunes are noted; turn the cylinder, and the tune for which it is set is played; draw it out a notch, and it gives a second; and so you may go on, till the whole number of tunes noted on it have had their turn. This, happily enough, represents the change which appears to take place in the vertebral chord and its ganglions, on the metamorphosis of the caterpillar into the butterfly, and the sequence of new instincts which result from the change. But if we extend the comparison, we may illustrate by it the two spheres of organized beings that we find on our globe, and their several instinctive changes and operations. We may suppose each kingdom of nature to be represented by a separate cylinder, having noted upon it as many tunes as there are species differing in their respective instincts —for plants may be regarded, in some sense, as having their instincts as well as animals—and that the constant impulse of an invisible agent causes each cylinder to play, in a certain order, all the tunes noted upon it: this will represent, not unaptly, what takes place with regard to the development of instincts, in the vegetable and animal kingdoms; and our simile will terminate in the enquiry, whose may be that invisible hand that thus shakes the sistrum of Isis,† and produces that universal harmony of action, resulting from that due intermixture of concords and discords, according to the will of its Almighty Author, in that infinitely diversified and ever-moving sphere of beings which we call *nature*."‡ 244.

And, pursuing the ingenious speculation of Dr. Virey, our author continues :—"What if these powers (celestial), employed as they are, by the

* Dr. Virey. † The Sistrum of Isis symbolized the elements.

Deity, to effect his almighty will, should also be the intermediate agents which, by their action on plants and animals, produce every physical developement and instinctive operation—*unless where* God *himself decrees a departure from any law that he has established?*" Such are the vague, we had almost said the silly and superstitious, hypotheses in which men indulge, when they attempt to unravel *final* causes ! ! To the credit of the medical profession, its cultivators have long abandoned such fruitless speculations, and confined themselves to such investigations as are within the sphere of man's capacities. The allusion to God's *departure* from his own established laws, is not very suitable to the understanding of the nineteenth century. Even in the mephitic air of Rome, at the present time, such *departures* are classed pretty generally with the miracles of saints, and the liquefaction of the blood of Januarius. These *departures* are attended with this bad effect—that their *interpretation* is always claimed by man—each sect of religionists interpreting them according to their own tenets and creed! Thus, every *unusual* phenomenon is accounted a *departure* from the laws of God, and a divine interposition ! We all know that the *cholera* was one of these *departures*, and that it was pronounced, from a thousand pulpits, as a direct visitation of Providence on a guilty world ! From this great example down to the descent of a thunderbolt, or even the upsetting of a coach, every striking event is an interposition of the Deity !

But to return. Our author appears to come to the conclusion that, as " the immediate cause of the vegetable instinct is clearly *physical*, so may be that of the animal." Where the actions of the animal are the result of intellect, then, of course, the cause is within the animal. We apprehend that this doctrine of Mr. Kirby will be looked upon with some degree of distrust, or even alarm, by some religionists. We think it trenches closely, though undesignedly, upon materialism. But we may wisely leave these idle speculations, and come to more practical and tangible matters.

Instincts may be divided into three general heads :—1st. Those relating to multiplication of the species, including the care of progeny—2d, the collection of food—3d, hybernation.

The pairing of animals usually begins in Spring—when the sun is beginning to gain power, and vegetation is beginning to come forth. This certainly looks like physical, rather than metaphysical excitement. But the preparation for the young, and the affection of the parent for the progeny, cannot be so easily accounted for. Our author's objection to a metaphysical impress, rather than to a physical one, founded on the mistake of the duck in hatching the young ducklings, is, as we have observed before, a very weak argument against instinct, as the direct impress of the Creator. The following is very little stronger.

" In truth, with regard to the higher animals, many associations may take place between the child and parent that help to endear the former to the latter. In the first place, the very circumstance of its being the fruit of her own bowels, and fed with milk from her own breast, must bind it to her by the tenderest of ties ; especially as, at the same time, it relieves her from what is troublesome. There is something also in infant helplessness, and infant gambols, calculated to win upon the doting mother. The subsequent alienation and estrangement of the female from her young, which takes place in all animals except man, appears, in the first instance, to be produced by their becoming troublesome and annoying to her ; which, in some degree, may account for her desire to cast them off." 259.

To us the above appears a "weak and impotent conclusion." It attributes to physical causes what, we are confident, is *instinct* implanted in the animal by the hand of the Creator. We rejoice to see a DIVINE so *liberal*, on these points; and we think we are entitled to some credit with the Church for being so *divine*, where materialism is so often charged to our account. But granting all that our author states in the above extract to be the result of purely physical causes, how will it explain the wearisome incubation of the hen, for weeks together, and every day turning her eggs in order that both sides may have an equal portion of the animal heat from the animal's body? We are totally at a loss to explain this by any physical impulse or impress. It is the impress of the Deity.

The migration of birds is more debateable. Mr. K. thinks it is "altogether physical." We doubt it. What is it that directs the swallow in its flight, and enables it to return to the very house or wall where it nestled the preceding Summer? The nidification of birds is also a most wonderful instinct.

Of the reptiles and fishes we know but little in these respects—except that they do not appear to feel that instinctive love for their young, after birth, exhibited by quadrupeds and birds. They have instinct enough, however, to select proper places to deposit their eggs, where they can be hatched by artificial or solar heat.

"Those of some *Ophidians*, as snakes, are buried in sand, and not seldom even in heaps of fermenting manure; while those of venomous ones are hatched in the womb of the dam, and come forth in the serpentine form. The *Saurians* also select a proper place for their eggs, and then desert them; the crocodile buries hers in the sands near the river; where many, however, are devoured by the ichneumon, and its other enemies, and are even relished by man. In the *Batrachian* Order one species of *salamander** commits a single egg to a leaf of the *Persicaria*, which it protects by carefully doubling the leaf, and then, proceeding to another, repeats the same manœuvre, till her oviposition is finished: the *toads* and *frogs* lay their eggs in the water, the former producing two long strings resembling necklaces, formed, as it were, of beads of jet, inclosed in crystal; while those of the latter consist of irregular masses of similar beads. This gelatinous or transparent envelope forms the first nutriment of the embryo. The nuptial song of the Reptiles is not, like that of birds, the delight of every heart, but is rather calculated to disturb and horrify than to still the soul. The hiss of serpents; the croaking of frogs and toads; the moaning of turtles; the bellowing of crocodiles and alligators, form their gamut of discords." 265.

The general object of migration in fishes appears to be the casting of the spawn. The salmon leaves the sea for the rivers—the herring travels South —the mackarel seeks the North—"all of them guided by an *indomitable* instinct." This very expression of our author is an admission that *physical* causes are inadequate to an explanation of the phenomena presented in these cases.

The numerous tribes of the insect world have all their seasons, varying according to their several destinies for fulfilling the great law of Nature, and to which the organization of each species is adapted. When the period for laying their eggs is arrived, each is impelled by instinct, and not by ob-

* Salamandra platycauda.

servation, to deposit them in a place where the young may find appropriate nourishment.

" From the instance of the flesh-fly, above related, we learn that it is their *scent* that directs insects to a proper station for their eggs." 266.

This does not solve the difficulty. What enables them to know, by means of this scent, which is the proper place of oviposition? GOD. Instinct.

FOOD. The different modes by which animals procure their food, afford the most striking proofs of creative wisdom in adapting the means to the ends.

" If we consider the infinite variety of substances, animal and vegetable, produced from the earth, which form the nutriment of its inhabitants—some solid and not easily penetrable; others soft and readily severed and comminuted; others again fluid, or semi-fluid;—we may conceive what a vast diversity of organs is necessary to effect this purpose. To render solid food, of any kind, fit for deglutition and digestion, the same mouth must be furnished with several kinds of teeth, some for incision, others for laceration, others again for grinding and mastication—while those that only absorb liquids merely require an organ adapted for suction, though often, at the same time, fitted to pierce the substance from which the nutritive fluid is to be derived." 267.

Our author believes that the fox has the power of *fascinating* the poultry, and can, in no other way, account for the manner in which he depopulates the hen-roosts by night. *Credat Judæus!* Of all animals, however, *insects* afford the most numerous instances of instinctive proceedings in this respect—witness the pitfalls of the ant-lion—the webs and nets of the spiders, &c. Many of them, however, require no stratagems, being endowed with sufficient force—witness the prominent eyes, tremendous jaws, and powerful wings and limbs of the tiger-beetle. A numerous tribe of insects that derive their nutriment from animals, merely deposit their eggs upon them—and thus, every bird that wings the air, every beast that walks the earth, every fish that swims the waters—even man himself, are infested in this way.

*Hybernation.* We shall not dispute the point of *physical* causes with our author here.

### ARACHNIDAN CONDYLOPES.

Our author now quits speculation, and returns to natural history. No animals fall more frequently under observation than spiders. We see them every where fabricating their snares and lying in wait for their prey. We observe them ascend into the air without wings, and carried along by their webs, as by an air-balloon! The wonderful apparatus for producing the materials of their webs is thus concisely described.

" At the posterior extremity of the abdomen, formed usually by a prominence, is the anus, immediately below which, planted in a roundish depressed space, are four or six jointed teat-like organs, of a rather conical or cylindrical shape. The exterior pair is the longest, consisting of three joints; but these have no orifices at their extremity for the transmission of threads: the other four* con-

* Mammulæ, Introd. to Ent. iii. 391.

sist each of two joints, and are pierced at their extremity with innumerable little orifices, in some species amounting to a thousand from each, from which their web issues at their will, or bristled with an army of infinitely minute biarticulate spinnerets,* each furnishing a thread at their extremity. These teats are connected with internal reservoirs, which yield the fluid matter forming the thread or web. These reservoirs in some species consist of *four,* and in others of *six* vessels folded several times, and communicating with other vessels in which the material that forms their web is first elaborated." 284.

The threads, after they issue from these organs, are united or kept separate, according to the will or the wants of the animal—and certain spiders can spin three different kinds of silk. The ordinary threads are about 24 times finer than those of the silk-worm—and yet one single thread will bear a weight six times that of the spider that weaves it! They employ their webs for three purposes—the construction of their snares—their own habitations—and the cocoon to contain their eggs. Some species have remarkable talent for architecture, hollowing out dens, boring galleries, elevating vaults, constructing subterraneous bridges, and forming doors that work upon hinges, and only want locks to make them equal to the same conveniences in the habitations of man! The interior of their houses is remarkable for neatness. Whatever be the humidity of the soil, water never penetrates them. The walls are covered with silken tapestry, presenting the lustre and gloss of satin. The bee and the ant furnish our author with ample illustrations of instinct, as well as the wonderful provision which God has made for all his creatures. We pass over a great space, and then mount another great link in the chain of creation.

### Fishes.

Hitherto we found no internal jointed vertebral column with its bony appendages, permeating spinal marrow—and brain, the most wonderful of all organs, the sensorium of feeling and the seat of the will. A brain and spinal marrow became necessary to animals, as they increased in size, and became "endowed with gradully increasing intellectual faculties." Fishes are placed by most naturalists as the basis of the vertebrated sub-kingdom. They are characterized by body, with vertebral column, covered with scales, moved by fins, respiration by permanent gills, one ventricle in the heart, blood red and cold. They are distinguished from birds and beasts by their breathing water instead of air. In this class the sense of smell is the most acute of all the senses. By it they can scent their prey at immense distances, through the deepest darkness and most agitated ocean. The olfactory nerve of the shark is expanded over a membrane twelve or thirteen feet square! Many of their parts, as the fins, if mutilated, can be reproduced. They are long-lived animals. A pike was taken in 1754, with a ring bearing date, 1487—or 267 years. It was nineteen feet long and weighed 350 pounds. Their emission of phosphorescent light, both while living and dead, is inexplicable, and its use unknown. Their fecundity is at least a million of times greater than that of land animals, and if there was no check by mutual depredations, they would fill the ocean, die, and corrupt the

* Fusi, ibid. 392.

air by putrefaction. Hence the utility of sharks and other sea monsters that swallow hundreds of the smaller fishes at a mouthful. The scene, it must be confessed, is one of blood and slaughter; but as the Creator is merciful, he has endowed fishes with little sensibility, and therefore they suffer infinitely less in death, than we imagine. The following argument will create a smile on the countenance of the philosopher.

"Another lesson may be learned from the existence of these terrible monsters; for if God fitted them to devour, he fitted them also to instruct. The existence of creatures so evil, and such relentless destroyers of his works in the material world, teach us that there are probably analogous beings in the spiritual world; and what occasion we have for watchfulness, to escape their destructive fury." 387.

What does Mr. Kirby mean by the *spiritual* world? We have need enough of watchfulness in this *visible* world, to escape the *sharks* and other rapacious monsters; but how our watchfulness can secure us against the "relentless destroyers" of the invisible world, we do not know.

It has been found very difficult to classify fishes—Linnæus and Cuvier not being agreed. Lacepede is the prince of modern icthyologists, and our author seems inclined to approve his classification, but follows that of Cuvier, according to the last edition of the "*Règne Animale*." We know comparatively little of fishes—except the best modes of catching and cooking them. We cannot dive into the depths of the ocean to investigate their manners and history; but we may take for granted that their instincts are as curious, and as demonstrative of the wisdom and goodness of the Creator, as those of terrestrial and aerial animals. We may, however, lightly allude to some curious particulars that are ascertained in respect to this interesting class of animated beings—so conducive to the support of the human race.

Among the Cartilagineans the HAG is remarkable for the singular means of escape from its enemies. It adheres to fishes by means of a sucker, formed of its lips. The adhesion effected, it lacerates the fish with its teeth, without their being able to shake it off, and then sucks their blood like a leech. But, in this situation, it would be liable to the attacks of other animals, were it not enabled to conceal itself by a copious effusion of excrement, which serves as an effectual protection during its depredation on its victim!

But among the diversified faculties, powers, and organs with which God has gifted various animals for warfare or defence, none is more wonderful than the electric batteries possessed by some fishes. They are, as it were, endowed with the lightning of Heaven, and enabled to employ an element more potent than gunpowder, to astound, smite, stupify, or kill the inhabitants of the waters. The faculty of the torpedo was known to Aristotle and Pliny. The Arabians observed this power of the torpedo and malapterurus, and seeing its affinity to the electric fluid of the clouds, called it *Raash*, or thunder.

"The *Torpedo* is the most celebrated of the electric fishes. In this the organ of its power extends, on each side, from the head and gills to the abdomen, in which space it fills all the interior of the body. Each organ is attached to the parts that surround it, by a cellular membrane and by tendinous fibres. Under the skin which covers the upper part of these organs, are two bands, one above the other, the upper one consisting of *longitudinal* fibres, and the lower of *transverse* ones. The latter continues itself in the organ by means of a great number of membranous elongations, which form many-sided vertical bodies, or hollow polygonal tubes, some hexagonal, others pentagonal, and others quadrangular; each of these tubes is divided, internally, by a fine membrane into several dissepiments, connected by blood-vessels. In each of the organs, from two hundred to twelve hundred of these tubes have been counted in individuals of different age and size, some regular but others irregular, which may form electric batteries. Each organ is also traversed by arteries, veins, and nerves, in every direction, which last are remarkable for their size." 398.

The two surfaces of the electric organ are supposed to be positive and negative. Almost always concealed in the mud these torpedos can kill the small fishes that come within their reach, or benumb the large ones. When attacked by the enemy, they can disable him by invisible blows. The Gymnotus Electricus has ten times more power than the Torpedo.

"It is stated that when the animal is touched with only one hand the shock is very slight; but when two hands are applied at a sufficient distance, a shock is sometimes given so powerful as to affect the arms with a paralysis for many years." 402.

"Humboldt gives a very spirited account of the manner of taking this animal, which is done by compelling twenty or thirty wild horses and mules to take the water. The Indians surround the basin into which they are driven, armed with long canes, or harpoons; some mount the trees whose branches hang over the water, all endeavouring by their cries and instruments to keep the horses from escaping: for a long time the victory seems doubtful, or to incline to the fishes. The mules, disabled by the frequency and force of the shocks, disappear under the water; and some horses, in spite of the active vigilance of the Indians, gain the banks, and overcome by fatigue, and benumbed by the shocks they have encountered, stretch themselves at their length on the ground. There could not, says Humboldt, be a finer subject for a painter: groups of Indians surrounding the basin; the horses, with their hair on end, and terror and agony in their eyes, endeavouring to escape the tempest that has overtaken them; the eels, yellowish and livid, looking like great aquatic serpents, swimming on the surface of the water in pursuit of their enemy." 403.

In a few minutes two horses were drowned. One of the electric eels (more than five feet long) gliding under the belly of a horse, discharged its battery on the region of the heart, and repeated the assault The animal, stupified by the reiterated shocks, fell into a profound lethargy, and sunk under the water, where it was drowned.

These formidable *gymnoti*, however, are soon exhausted by their electric discharges, and become harmless, till their powers are recruited by rest.

"Swimming half out of the water, they flee from the horses instead of attacking them; and if they enter it the day after the battle, they are not molested, for these fishes require repose and plenty of food to enable them to accumulate a sufficient supply of their galvanic electricity." 403.

The *fishing-frog*, or sea-devil, as it is sometimes called, presents a remarkable instinct. It is often six or seven feet in length, yet it has neither defensive arms, nor force in its limbs, nor celerity in its movements. It makes up for the want of all these by cunning and stratagem. It plunges in the mud, or conceals itself among stones or sea-weed, letting no part be seen except the filaments that fringe its body. These appearing like worms, form a kind of bait by which the fishing-frog allures other fishes within its grasp, when it suddenly swallows them down its enormous throat!

We had hoped to conclude our Review of Mr. Kirby's work in this article; but find that it would require too much space. In our next number we shall dedicate ten or a dozen pages to reptiles, birds, and mammalians.

